# Social Inequalities in Long-Term Health Effects After COVID-19—A Scoping Review

**DOI:** 10.3389/ijph.2024.1606739

**Published:** 2024-02-07

**Authors:** Nina Lammers, Florian Beese, Jens Hoebel, Christina Poethko-Müller, Benjamin Wachtler

**Affiliations:** Department of Epidemiology and Health Monitoring, Robert Koch Institute, Berlin, Germany

**Keywords:** post-COVID-19 condition, long COVID, socio-economic position, social inequalities, scoping review

## Abstract

**Objectives:** We aimed to map and synthesize evidence about social inequalities in long-term health effects after COVID-19 (LTHE), often referred to as “long COVID” or “post-COVID-19 conditions.”

**Methods:** We conducted a scoping review of peer-reviewed articles by searching the databases Embase and Scopus. According to predefined inclusion criteria, titles/abstracts and full texts were screened for eligibility. Additionally, reference lists of all included studies were hand-searched for eligible studies. This study followed the PRISMA guidelines for scoping reviews.

**Results:** Nineteen articles were included. LTHE were analysed according to ethnicity, education, income, employment and deprivation indices. The studies varied significantly in their definitions of LTHE. Eighty-two analyses showed no statistically significant associations. At least 12 studies had a high risk of type II errors. Only studies associating deprivation indices and long COVID tended to show a higher prevalence of LTHE in deprived areas.

**Conclusion:** Although some studies indicated social inequalities in LTHE, evidence was generally weak and inconclusive. Further studies with larger sample sizes specifically designed to detect social inequalities regarding LTHE are needed to inform future healthcare planning and public health policies.

## Introduction

It has been estimated that well over 200 million individuals globally did not recover to their prior health status (restitutio ad integrum) after their acute COVID-19 infection [1]. This high number of estimated post-COVID-19 cases was published on 16 April 2022. Since then, further millions of people worldwide have been infected with SARS-CoV-2, and the number of individuals affected by long-term effects of COVID-19 can be considered to be much higher, as recent population-based cohort studies estimate that at least 6.2% of SARS-CoV-2 patients do not recover from the acute infection [2, 3]. Affected individuals suffer from persistent or newly occurring symptoms months after their infection with COVID-19. A wide range of heterogeneous long-term symptoms have been reported in the literature to date, including fatigue/exhaustion and cardiopulmonary, neurological, gastrointestinal, cognitive and psychological conditions [1, 4–10]. Long-term symptoms generally impact the everyday functioning of affected individuals, who often require long-term treatment and rehabilitation and are sometimes unable to continue working. In addition to individual suffering, long-term symptoms are considered a burden for national health and social systems as well as the economy [11, 12].

In December 2020, the United Kingdom’s National Institute for Health and Excellence (NICE) published an initial guideline on the long-term health effects of COVID-19, and distinguished between “ongoing symptomatic symptoms” (symptoms from 4 to 12 weeks after acute infection) and “post-COVID-19 syndrome” (new or lasting symptoms 12 weeks or more after acute infections), stating that both—“ongoing symptomatic symptoms” and “post-COVID-19 syndrome”—are commonly described by using the term “long COVID” [13].

About 10 months later, in October 2021, the World Health Organisation (WHO) released a clinical case definition naming the long-term effects of COVID-19 “post-COVID-19 condition” (PCC), defining PCC as follows:

Post-COVID-19 condition occurs in individuals with a history of probable or confirmed SARS-CoV-2 infection, usually 3 months from the onset of COVID-19 with symptoms that last for at least 2 months and cannot be explained by an alternative diagnosis. […] Symptoms might be new onset after initial recovery from an acute COVID-19 episode or persist from the initial illness. Symptoms might also fluctuate or relapse over time [14].

Today, most research refers to either the NICE guideline or the WHO clinical case definition. Both definitions are considered provisional as knowledge regarding the aetiology, risk factors, clinical treatment and rehabilitation of long COVID/PCC is still progressing. As long COVID/PCC is an evolving clinical entity and further research on biomarkers to confirm the condition is needed [15], it is still a diagnosis of exclusion. To date, no clear pattern of risk factors for post-COVID-19 condition has been identified. While the risk of long-term effects appears to be higher in COVID-19 patients with severe acute infections [16] and those with comorbidities [17], long COVID/PCC has also been diagnosed in individuals with mild acute infections [16] and previously healthy individuals [18]. Therefore, it is considered that anyone infected with SARS-CoV-2 is at risk of long COVID/PCC [19, 20].

According to recent study findings, vaccinations against SARS-CoV-2 have demonstrated protective effects. Vaccinated COVID-19 patients had a lower risk of developing long COVID/PCC after breakthrough infections than unvaccinated individuals [15, 17, 21–23]. However, due to the heterogeneity of these studies, the extent to which vaccinations offer protection remains unclear. Furthermore, an indirect protective effect of vaccinations can be assumed as they reduce the risk of infection and the risk of severe acute infections [24].

Research has shown that social determinants are associated with vaccination rates [25, 26] and with infection rates throughout the different stages of the pandemic [27]. Therefore, we assume that the risk factor for long COVID/PCC (infection with SARS-CoV-2) and the protective factor (vaccination) have not been equally distributed either, which may have resulted in different outcomes of long-term effects of COVID-19 for individuals from different social positions. Systematic research on social inequalities and its potential associations with long-term health effects of COVID-19 is still scarce. But knowledge about social inequalities in long COVID/PCC may be of great importance for public health measures. If it were found that long-term symptoms were associated with social determinants, particularly affected groups could be addressed specifically. For instance, this could involve facilitating access to medical experts and relaunching vaccination campaigns as a preventive measure.

We therefore conducted a scoping review to collate and initially map the available evidence on social inequalities regarding the long-term health effects of COVID-19. This study aimed to answer the research question of what is already known about social inequalities in long-term health effects of COVID-19 and the following three subquestions: 1. “How do researchers define ‘socio-economic position’ and ‘long-term effects of COVID-19’ in the current research into social inequalities in long COVID/PCC?” 2. “What study designs are used to answer this research question?” 3. “What effect estimators are used to measure the association between socio-economic position and long-term health effects of COVID-19?”

## Methods

We conducted a scoping review to answer the research question. Scoping reviews serve as a type of knowledge synthesis to systematically “map evidence on a topic and identify main concepts, theories, sources, and knowledge gaps” [28]. We used the “Population, Concept, Context” framework [29] to further specify our research questions. The population of interest was restricted to the general population, the concept was social inequalities in long-term health effects of COVID-19 and the context was left open as sources from any contextual setting, such as high-income countries as well as low- and middle-income countries, were eligible for inclusion [29].

We followed the Preferred Reporting Items for Systematic Reviews and Meta-Analyses Extension for Scoping Reviews (PRISMA-ScR), which is based on Arksey and O’Malley’s theoretical framework and Levac et al.’s and Peters et al.’s further developments and updates [28–31]. A publicly accessible study protocol was registered on 12 September 2022 [32].

### Search Strategy

We searched the databases Embase and Scopus on 14 September 2022. Both search strings are provided in the Supplementary Material. Results were exported to EndNote, where duplicates were removed, and then exported to Rayyan, a software that supported the selection process. The selection process was conducted by two reviewers independently (NL and FB) in a two-step approach. In case of conflict between the two reviewers, a third member of the research team made the decision about inclusion or exclusion (BW). First, articles were screened by title and abstract. If they met the inclusion criteria, a full text screening followed. Additionally, the reference lists of included articles were hand-searched. Interrater reliability was determined via Cohen’s Kappa coefficient for both stages using the statistical software R (version 4.0.4).

### Eligibility Criteria

We included studies that focused on general populations on at least the regional level and excluded studies that focused on specific subpopulations such as hospitalized individuals etc. to minimize the risk of collider bias [33]. We included empirical quantitative analyses that linked socio-economic measures on the individual or the area level to long-term health effects of SARS-CoV-2 infection. Anglo-American research on social inequalities of acute COVID-19 infections often used the variables “race/ethnicity” to measure health disparities in infection rates [34–36]. To map social inequalities in long-term health effects of COVID-19 we also included studies that linked the variables “race/ethnicity” with long-term health symptoms of COVID-19. To avoid biological connotations, “race/ethnicity” had to be contextualized with social and/or socio-economic factors when they were operationalized by research other than Anglo-American studies, where the concept “race/ethnicity” is commonly contextualized in interaction with social position [37, 38].

In the early stages of the pandemic, consistent definitions for long-term health effects of COVID-19 were not yet established, leading us to include all relevant concepts. Due to the heterogeneity of the included studies, a structured risk of bias assessment could not be conducted. To ensure a certain level of scientific quality, we included peer-reviewed articles only. Only German and English articles were included. Reviews were screened for further eligible articles and then excluded. All inclusion and exclusion criteria are presented in Table 1.

**TABLE 1:** Eligibility criteria for the study selection (Germany, 2023).

	Inclusion	Exclusion
Population	• General populations	• Subpopulations (e.g., with certain health conditions, etc.)
Study design	• Empirical quantitative studies with own data-analyses (cross-sectional, case-control, cohort studies, ecological studies)	• Theoretical research
	• Scoping or systematic reviews will only be screened for their included articles	• Studies without own data-analyses
		• Qualitative studies
Publication type	• Peer-reviewed articles	• All other
	• References in included articles that fulfill the inclusion criteria	
Long-term effects of COVID-19	• All concepts of long-term health effects of COVID-19	—
Social determinants	• Social determinants measured on the individual and on the area level	• Race/ethnicity not contextualized with social position
Outcome	• Measures of association between long-term health effects of COVID-19 and social determinants e.g., incidence, prevalence, measures of risk (risk ratio, odds ratio, hazard ratio etc.)	• Crude cases without denominators
Language	• English and German	• All other

### Data Charting and Synthesis of Results

A PRISMA flow chart was used to visualize and summarize the selection process. Data from the included studies were extracted by using a standardized data extraction chart. We extracted the following information: publication year, country, study design, sample size, definition and duration of long-term effects, social and socio-economic measures, effect estimators, outcomes, and associations between social determinants and long-term effects for each study. Associations between socio-economic position (SEP) and long-term health effects were categorized as 1) “lower SEP more affected”; 2) “higher SEP more affected”; and 3) “no association”. “Lower SEP more affected” was assigned if statistically significant results showed that people from a lower SEP were more affected by long-term health effects of COVID-19, and “higher SEP more affected” was used when statistically relevant results showed that people from a higher SEP were more affected by long-term health effects of COVID-19. Associations between race/ethnicity and long-term health effects were categorized as 1) “More affected” or 2) “Less affected,” compared with White/Non-Hispanic White study participants, and 3) “No association.” “No association” was used if the analyses of social determinants and long-term health effects of SARS-CoV-2 infection provided no statistically significant results. Statistical significance was denoted if a *p*-value of < 0.05 was provided or—in case of ratios—the provided 95% confidence intervals (CIs) did not contain the “1”. Regarding presented rates, results were considered significant if CIs did not overlap [39].

Results were summarized descriptively through tables and figures and then further synthesized narratively [40]. Social determinants were distinguished according to their structures and categorized as “nominal” and “ordinal”. Two approaches were used to assign the ordinal SEP measures to one of the three associations: first, a result was assigned to “lower SEP more affected” or “higher SEP more affected” if results at all ordinal levels were statistically significant. As this approach carried the risk of missing significant results, it was followed by the second approach in which results were categorized as “lower SEP more affected” or “higher SEP more affected” if statistically significant analyses could be observed at at least one ordinal level. This allowed for both a lower and an upper bound of statistically significant results to be identified.

## Results

**FIGURE 1:**
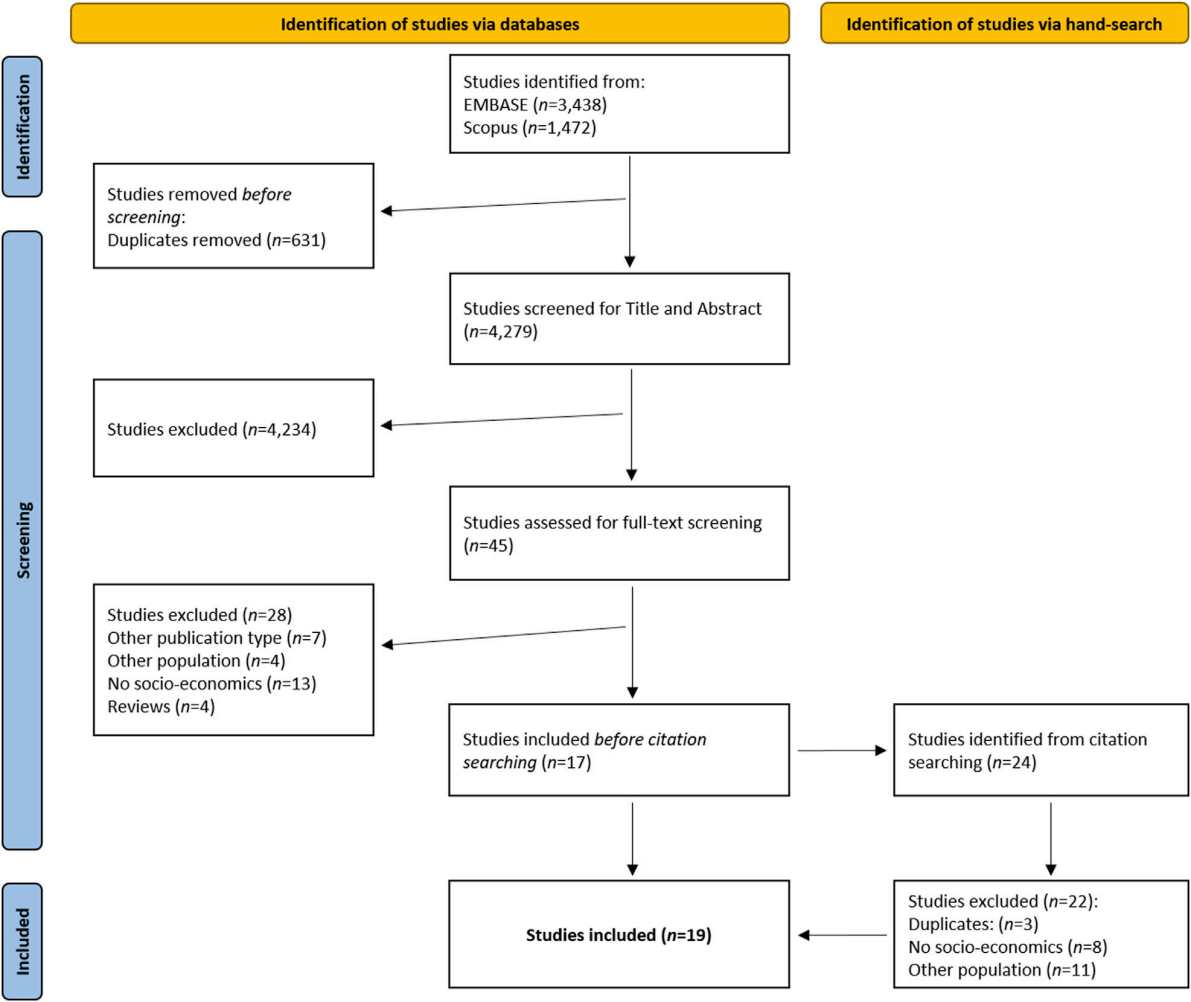
Flow chart of the study selection process, based on the Preferred Reporting Items for Systematic reviews and Meta-Analyses extension for Scoping Reviews (PRISMA-ScR) guideline (Germany, 2023).

We included 19 articles that met all inclusion criteria, most of which presented data from the United States of America (*n* = 7) and the United Kingdom (*n* = 5). The PRISMA flow chart in Figure 1 provides a detailed overview of the selection process.

The interrater reliability was high, with Cohen’s kappa coefficients of 0.79 for the title and abstract screening and 0.91 for the full-text screening. The extracted data for each study is presented in Table 2.

**TABLE 2:** Summary of findings (Germany, 2023).

Study	Country	Study design	Sample size (LC cases^a^)	Definition “long-term health effects”	Social determinants	Estimator	Association*
Abdelhafiz et al. (2022) [41]	Egypt	Cross-sectional	396 (347)	One or more self-reported symptoms ≥3 weeks after acute infection	Education	OR	No association
Abdelwahab et al. (2022) [42]	USA	Longitudinal	19,792 (8,290)	Diagnosed PACS (Postacute Sequelae of SARS-CoV-2) symptoms, not present before COVID, ≥4 weeks after acute infection	Race/ethnicity	OR	Black: No association
							Asian: More affected^b^
		Retrospective analysis of health records					Hispanic: No association
							Other: No association
							Declined: No association
AlRadini et al. (2022) [43]	Saudi Arabia	Cross-sectional	1,000 (317)	One or more self-reported symptoms ≥4 weeks after acute infection; symptoms: list categorized by subject matter experts	Employment	OR, aOR	No association
Förster et al. (2022) [44]	Germany	Cross-sectional	1,459 (715)	One or more self-reported symptoms ≥12 weeks after acute infection	Education	OR	Higher SEP more affected
Frontera et al. (2021) [45]	USA	Cross-sectional	999 (19)	One or more self-reported symptoms ≥4 weeks after acute infection; symptoms: listed by CDC	Race/ethnicity	Proportions	Black: More affected^b^
							Other: More affected^b^
					Education		Hispanic: More affected^b^
							Education: No association
Hirschtick et al. (2021) [46]	USA	Cross-sectional	593	Self-reported return to usual state of health after COVID ≥4 weeks and ≥8 weeks after acute infection	Race/ethnicity	aPR	Hispanic ≥4: No association
							Non-Hispanic Black ≥4: No association
							Non-Hispanic Other ≥4: No association
							Hispanic ≥8: No association
					Income		Non-Hispanic Black ≥8: No association
							Non-Hispanic Other ≥8: No association
							Income ≥4: No association
							Income ≥8: No association
Hutchinson et al. (2022) [47]	UK	Cross-sectional	945,000	One or more self-reported symptoms ≥4 weeks after acute infection	IMD	PR	Lower SEP more affected
		Projection of statistical data					
Islam et al. (2021) [48]	Bangladesh	Cross-sectional	1,002 (103)	Self-reported COVID-like symptoms after COVID-19 stage	Income	aOR	No association
John et al. (2022) [49]	India	Cross-sectional	782 (377)	Self-reported fatigue and sleep disorders from early infection period ≤12 weeks after acute infection	Education	Proportions	Education: No association
				Fatigue = Fatigue Assessment Scale (FAS) ≥22	Income		Income: No association
				Sleep disorder = sleep quality score (SQS) 57–84	Employment		Occupation: No association
Office for National Statistics (2021) [50]	UK	Cross-sectional	21,622	Ongoing symptomatic (OGS): 4–12 weeks after acute infection	Race/ethnicity	PR	Black (OGS): No association
							Mixed (OGS): No association
							Asian (OGS): Less affected^b^
				Post-COVID-19 syndrome (PCS): ≥12 weeks after acute infection			Other (OGS): No association
							Black (PCS): No association
					IMD		Mixed (PCS): No association
				One or more self-reported symptoms unexplained by something else			Asian (PCS): Less affected^b^
							Other (PCS): No association
							IMD (OGS): Lower SEP more affected
							IMD (PCS): No association
Ogungbe et al. (2022) [51]	USA	Cross-sectional	442 (190)	One or more self-reported symptoms of cardiac-related PASC ≥3 after acute infection	Race/ethnicity	OR, aOR	Black: No association
							Asian: No association
					Education		Hispanic: No association
				Cardiac-related PASC = sudden chest pain, tachycardia, higher than normal pulse rate, faintness, heart palpitations			Other: No association
					Income		Education: No association
							Income: No association
Stavem et al. (2021) [52]	Norway	Cross-sectional	440 (212)	Self-reported fatigue 6–24 weeks after acute infection	Education	OR	No association
				Fatigue = Chalder fatigue scale (CFQ)-11 bimodal score, >3 vs. 0–3			
Subramanian et al. (2022) [53]	UK	Longitudinal	384,137 (29,869)	One or more symptoms ≥12 weeks after acute infection, unexplained by alternative diagnosis	Race/ethnicity	HR, aHR	Asian (HR): Less affected^b^
							Black (HR): No association
							Mixed (HR): No association
							Other (HR): Less affected^b^
							Asian (aHR): No association
		Retrospective analysis of primary care data		Symptoms = symptoms previously reported to be associated with long COVID by epidemiological studies, patients, and clinicians	IMD		Black (aHR): More affected^b^
							Mixed (aHR): More affected^b^
							Other (aHR): More affected^b^
							IMD (HR): Lower SEP more affected
							IMD (aHR): No association
Tenforde et al. (2020) [54]	USA	Cross-sectional	270 (95)	Self-reported return to health 2–3 weeks after acute infection	Race/ethnicity	OR, aOR	Non-Hispanic Black: No association
							Non-Hispanic Other: No association
							Hispanic: No association
Thompson et al. (2022) [55]	UK	Longitudinal	1,068,680 (4,189)	Long COVID codes in health records	Race/ethnicity	OR	Mixed: No association
							South Asian: Less affected^b^
		Retrospective analyses of health records			IMD		Black: Less affected^b^
							Other: No association
							IMD: Higher SEP more affected
Thompson et al. (2022)^c^ [55]	UK	Longitudinal	6,907 (1,027)	One or more self-reported symptoms ≥4 and ≥12 weeks after acute infection	Race/ethnicity	OR	Non-White (≥4): No association
							Non-White (≥12 CM): Less affected^b^
					IMD	Common effect model (CM)	Non-White (≥12 RM): No association
		Meta-analyses of cohort studies					IMD: No association
					Education	Random effects model (RM)	Education (≥4): No association
							Education (CM & RM): Higher SEP more affected
Westerlind et al. (2021) [56]	Sweden	Longitudinal	8,916 (789)	Long COVID codes	Education	OR	Education: No association
					Income		Income: No association
		Retrospective analyses of health insurance data					Employment: Ref.
					Employment		Self-employment: More affected
							Unemployment: No association
Whitaker et al. (2022) [57]	UK	Cross-sectional	71,642 (31,558)	One or more self-reported symptoms ≥12 weeks after acute infection	Race/ethnicity	OR, aOR	Mixed (OR): More affected^b^
							Black (OR): No association
							Asian (OR): Less affected^b^
					Income		Other (OR): No association
				29 predefined symptoms			Mixed (aOR): No association
		Retrospective analysis of cohort that was interviewed at one time point					Black (aOR): No association
							Asian (aOR) Less affected^b^
					IMD		Other (aOR): No association
							Income: Lower SEP more affected
							IMD (OR): Lower SEP more affected
							IMD (aOR): No association
Whitaker et al. (2022)^d^ [57]	UK	Cross-sectional	13,170 (2,845)	One or more self-reported symptoms ≥12 weeks after acute infection	Race/ethnicity	OR, aOR	Mixed (OR): No association
							Black (OR): No association
							Asian (OR): Less affected^b^
					Income		Other (OR): No association
							Mixed (aOR): No association
		Retrospective analysis of cohort that was interviewed at one time point		29 predefined symptoms			Black (aOR): No association
							Asian (aOR) Less affected^b^
					IMD		Other (aOR): No association
							Income (OR): Lower SEP more affected
							Income (aOR): No association
							IMD: No association
Wu et al. (2022) [58]	USA	Longitudinal	308 (74)	One or more self-reported symptoms ≥12 weeks after acute infection	Race/ethnicity	OR	Non-Hispanic Black: No association
							Hispanic: No association
		Retrospective analysis of cohort that was interviewed three times: before, during and after COVID-19		Symptoms according to CDC	Education		Non-Hispanic Other: No association
							Education: No association
Yomogida et al. (2022) [59]	USA	Cross-sectional	363 (128)	One or more self-reported symptoms ≥8 weeks after acute infection	Race/ethnicity	aIRR	Asian: No association
							Black: More affected^b^
							Hispanic: No association
							Other: No association

OR, odds ratio; aOR, adjusted odds ratio; PR, prevalence ratio; aPR, adjusted prevalence ratio; HR, hazard ratio; aHR, adjusted hazard ratio; aIRR, adjusted incidence rate ratio; IMD, Index of Multiple Deprivation; UK, United Kingdom; USA, United States of America.

*According to statistically significant level of *p* < 0.05. Statistical significance was denoted when all categorical levels of ordinal variables showed significant results.

^a^ Numbers of long COVID cases were not presented by Hirschtick et al., who presented weighted row percentages, and Hutchinson et al. and the ONS, who estimated prevalence at populational levels.

^b^ Compared with White study participants.

^c^ Thompson et al. are listed twice, as their analyses were based on two different databases.

^d^ Whitaker et al. are listed twice, as they provided additional analyses of a subset that was used for sensitivity analyses.

### Study Characteristics

Fourteen studies had a cross-sectional design [41, 43–52, 54, 57, 59], while the rest (*n* = 5) followed a longitudinal design [42, 53, 55, 56, 58]. Twelve of the 14 cross-sectional studies conducted surveys, collecting their data at one time point only. Two studies worked with already existing survey data, which were also collected at one time point [47, 57]. Four of the five longitudinal studies analysed health records [42, 53, 55] and insurance data [56] over defined time periods. One study defined a subsample from an existing cohort study where the same individuals were surveyed three times: 1 month before, around the time of, and 12 weeks after infection [58].

The analyses of the association between social determinants and long-term health effects of SARS-CoV-2 infection were based on sample sizes varying from 270 [54] to 1,068,680 [55] with a mean of 121,330 and a median of 1,002, indicating that the majority of included studies had rather small sample sizes and that studies with large sample sizes were outliers. A tendency towards cross-sectional studies working with small sample sizes and longitudinal studies analysing mostly larger samples was noted.

Only one research team included non-COVID controls in their statistical analyses of the association between social determinants and long-term health effects [45]. All the other studies examined participants from COVID-19 cases and obtained their results by comparing study participants experiencing long-term symptoms after acute infection with participants who had recovered.

None of the included studies analysed the association between social determinants and long-term-symptoms as the primary research question.

### Social Determinants

The 19 included studies used five different measures to operationalize different social determinants, four on the individual level and one on the area level. At the individual level, the studies used data referring to race/ethnicity (*n* = 11), education (*n* = 9), income (*n* = 6) and employment (*n* = 3). On the area level, five studies operationalized social determinants by referring to indices of multiple deprivation (IMDs), which were all from the United Kingdom. Several studies used more than one social determinant. Race/ethnicity was only analysed by studies from the United States and the United Kingdom. A detailed overview of the used social determinants is presented in Table 3.

**TABLE 3:** Measure and number of social determinants used by the included studies (Germany, 2023).

	*n*	Studies
Race/ethnicity	**11**	[42, 45, 46, 50, 51, 53–55, 57–59]
White	8	[42, 45, 50, 51, 53, 55, 57, 59]
Non-Hispanic White	3	[46, 54, 58]
Black	8	[42, 45, 50, 51, 53, 55, 57, 59]
Non-Hispanic Black	3	[46, 54, 58]
Asian	7	[42, 45, 50, 51, 53, 57, 59]
South Asian	1	[55]
Hispanic	6	[42, 45, 46, 51, 54, 58]
Hispanic or Latino	1	[59]
Other	8	[42, 45, 50, 51, 53, 55, 57, 59]
Non-Hispanic Other	3	[46, 54, 58]
Mixed	5	[50, 53–55, 57]
Native American/Alaskan Native	1	[45]
Pacific Islander/Native Hawaiian	2	[45, 59]
Non-Hispanic	1	[45]
Non-White	1	[55]
American Indian	1	[59]
Unknown, declined	3	[42, 45, 59]
Income	**6**	[46, 48, 49, 51, 56, 57]
Annual household income (3 levels)	1	[46]
Monthly family income (3 levels)	1	[48]
Annual income	2	[49, 56]
Household income (4 levels)	2	[51, 57]
Education	**9**	[41, 44, 45, 49, 51, 52, 55, 56, 58]
Pre-university, university, post-university	1	[41]
No qualifications, school certificate, vocational training diploma, university degree	1	[44]
High school diploma, some college, bachelor’s degree, graduate degree	1	[51]
Years of education (median)	1	[45]
Educational level	1	[49]
Primary school <11 years, secondary school 12–13, university	1	[52]
No higher education, degree attained	1	[55]
Primary school, secondary school, short and long university education	1	[56]
High school and lower, some college, college and more	1	[58]
Employment	**3**	[43, 49, 56]
Unemployed, employed, retired, student, healthcare employed	1	[43]
Employment status	1	[49]
Employment, self-employment, unemployment	1	[56]
IMD (5 deprivation quintiles)	**5**	[47, 50, 53, 55, 57]

IMD, index of multiple deprivation.

Bold value indicate the number of studies that included this variable.

### Definitions of Long-Term Health Effects of COVID-19

**FIGURE 2:**
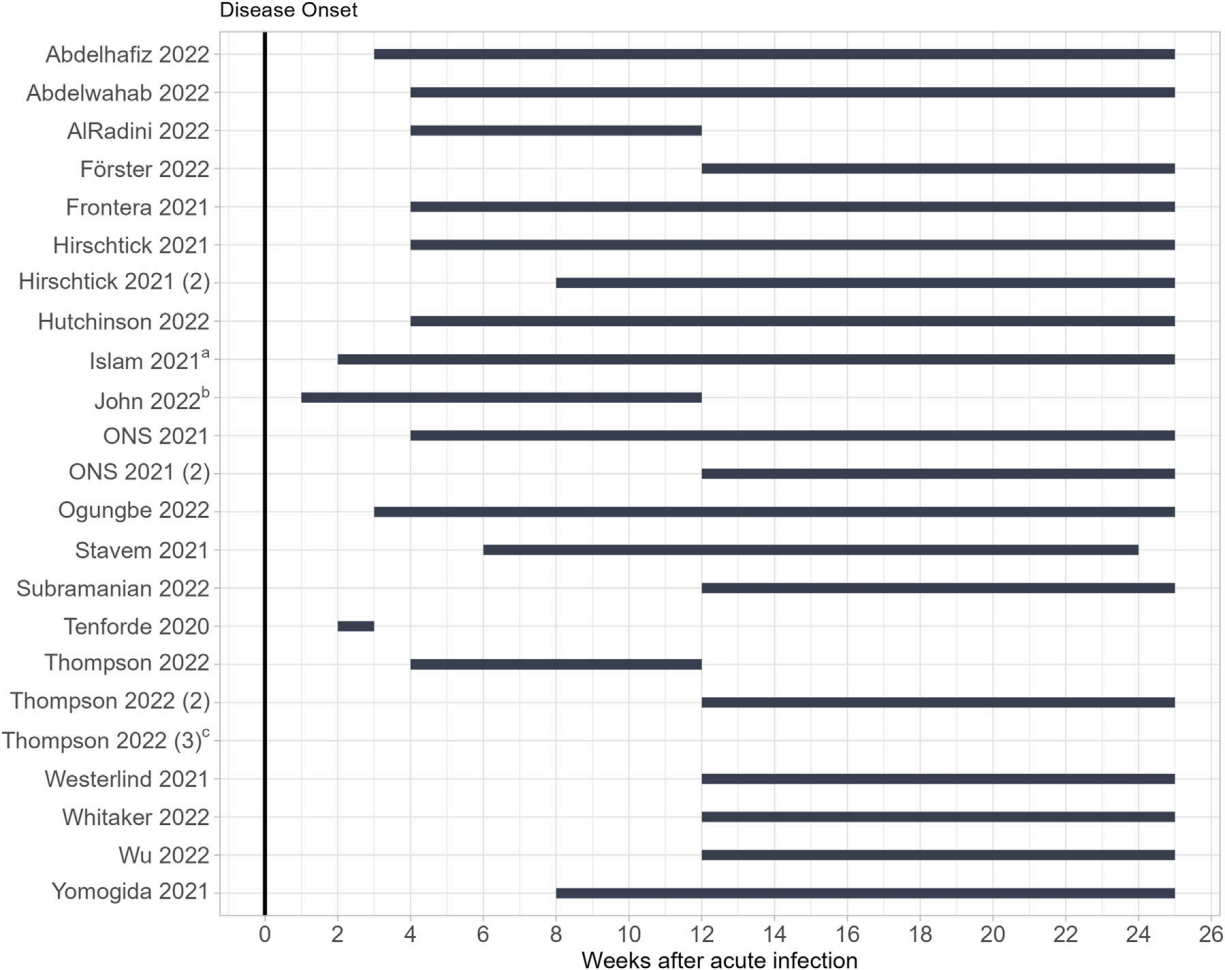
Observation periods of the included studies in relation to the acute COVID-19 infection (Germany, 2023). ^a^ Start denoted as “after COVID-19 stage”. ^b^ Start denoted as “early infection period”. ^c^ No time provided, analyses were based on long-COVID codes in electronic health records. ONS, Official for National Statistics.

The included studies varied in their definition of “post-COVID-19 condition”: five studies referred to the NICE guidelines [47, 50, 55, 57, 58], four based their analyses on the WHO clinical case definition [44, 53, 56], two referred to a temporal definition of the Centers for Disease Control and Prevention (CDC), that cannot be found online any more [41, 43, 45] and eight studies did not use any standardized evidence-based definition to operationalize long-term health effects of COVID-19 [42, 46, 48, 49, 51, 52, 54, 59]. Figure 2 shows observation periods of the included studies in relation to the acute COVID-19 infection.

Most of the studies measured long-term symptoms starting 4 weeks after acute infection (*n* = 7) or 12 weeks after acute infection (*n* = 7) which corresponds with the NICE guideline for long COVID [13] or the WHO definition for PCC [14].

Corresponding with the timings of the NICE guideline, Thompson et al. provided two analyses, and measured long-term symptoms 4–12 weeks after acute infection (the time frame for “ongoing symptomatic COVID-19”) and ≥12 weeks after acute infection (the time frame for “post-COVID-19 syndrome”) [55]. Hirschtick et al. also provided two analyses based on two different time measurements, which they labelled as “30+ day COVID-19” for symptoms persisting for 30 or more days and “60+ day COVID-19” for symptoms lasting for 30 or more or 60 or more days after acute SARS-CoV-2 infection [46]. Five studies measured symptoms earlier than 4 weeks after disease onset [41, 48, 49, 51, 54]. Fifteen included studies based their analyses on self-reported long-term health effects of COVID-19. Four studies relied on medically assessed long-term symptoms [42, 53, 55, 56].

### Outcomes and Statistical Measures

The included studies differed in their measurement of social inequalities in long-term health effects of COVID-19. Six studies presented predictors for long-term symptoms and estimated odds ratios [41–44, 57, 58]. One study also presented predictors but calculated adjusted incidence rate ratios [59]. Studies calculating risk factors for long COVID provided odds ratios, hazard ratios, or adjusted prevalence ratios. The Office for National Statistics (ONS) estimated stratified prevalence rates for the United Kingdom. Hutchinson et al. also estimated stratified prevalence rates, but only for England and based their estimations on a different time period than the ONS [47, 50]. Other studies estimated odds ratios related to specific long-term health effects according to the scope of these studies, e.g., odds for persistent fatigue or sleep disorders, cardiac-related PASC or sick leave due to long COVID [51, 52, 56]. Two studies reported chi-square test results for the association between social determinants and long-term health effects [45, 49].

### Associations between Social Determinants and Long-Term Health Effects of COVID-19

**FIGURE 3:**
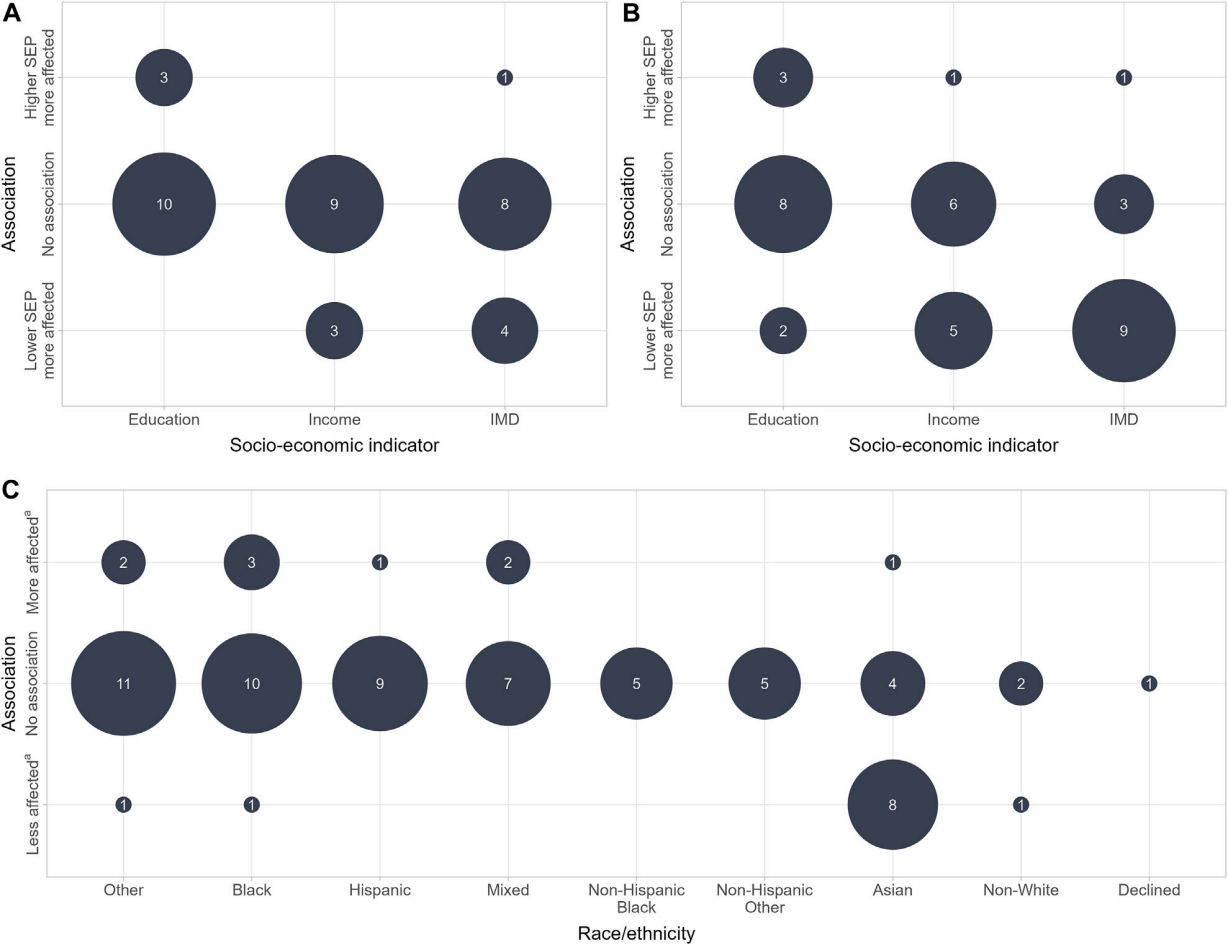
Associations between social determinants and long-term health effects of COVID-19 (Germany, 2023). **(A)** Significant association at all categorical levels. **(B)** Significant association at one categorical level at least. **(C)** Associations between race/ethnicity and long-term health effects of COVID-19. ^a^ Compared with White or non-Hispanic White study participants. SEP, socio-economic position; IMD, Index of Multiple Deprivation.

The 19 included studies analysed 74 associations between race/ethnicity, and the long-term health effects of COVID-19 (Figure 3) and 12 associations between employment and long-term health effects of COVID-19. The analyses of the ordinal SEP measures “education,” “income,” and “IMD” resulted in 38 associations with long-term health effects (Figure 3).

#### Ordinal SEP Measures

The ordinal SEP measures “education,” “income” and “IMD” were analysed 38 times. Based on statistically significant results on all categorical levels, 27 of the associations were categorized as “no association”; seven as “lower SEP more affected”; and four as “higher SEP more affected.” Based on the second assignment approach, the ordinal SEP measures “education,” “income,” and “IMD” were assigned to one of the three associations based on statistically significant results at one of the categorical levels at least, which led to all the possible combinations of the three associations with the ordinal SEP measures. Based on this approach, the most measured association analysing “IMD” and persistent symptoms was “lower SEP more affected” (*n* = 9). In the analyses of “education” and “income” and long-term health effects of COVID-19, “no association” remained the most frequent association (*n* = 8) (Figure 3).

#### Measures of Employment

Three studies analysed SEP measures with no hierarchical order, referring to measures of employment [43, 49, 56]. These studies provided 12 associations between measures of employment and the long-term health effects of COVID-19. All analyses were categorized as “no association,” except for the analyses conducted by Westerlind et al., who compared self-employed study participants with permanently employed individuals and provided higher odds for sick leave after COVID-19 for self-employed individuals [56].

#### Race/Ethnicity

Seventy-three per cent (*n* = 54) of the associations between race/ethnicity, and the long-term health effects were categorized as “no association.” Twelve percent (*n* = 9) of the analyses showed that long-term health effects affected Black, Hispanic, Asian, Mixed and individuals from other ethnicities more than White individuals. Fifteen percent (*n* = 11) of the analyses showed that long-term health effects affected Black, Asian, Mixed, Other and non-White individuals less than White individuals. “No association” was the most frequent outcome across all ethnic groups excepting Asian individuals, who were mostly found to be less affected by long-term health effects than White study participants. All studies coming to that result were from the UK and accordingly referred to Asians living in the United Kingdom.

## Discussion

The included studies did not provide a clear pattern of social inequalities in long-term health effects of COVID-19. Most of the included analyses resulted in the outcome “no association,” but the results carried a high risk of type II errors, owing to the small sample sizes that some of the included studies based their analyses on. Studies with large sample sizes that came to statistically significant results were mainly from the UK. They showed that individuals living in more deprived areas seem to be more affected by long COVID/PCC than those living in less deprived areas (Figure 3). A second finding from the UK studies was a lower affection by long COVID/PCC in Asian individuals compared with the White population in the United Kingdom (Figure 3) [47, 50, 53, 55, 57]. These studies were comparable due to a coherent use of the NICE guidelines and the WHO definition of PCC. Furthermore, the results seem to be robust due to large sample sizes and good representativeness. Further research is required to investigate reasons for the lower affection of Asians in the United Kingdom. In addition, only UK studies have found that the prevalence of long COVID/PCC could be associated with living in deprived areas. Whether or not this observation also applies to individuals living in deprived areas of other countries remains a research gap.

The included studies varied significantly in their definitions of “long-term effects of COVID-19,” with 42% not following any case definitions or guidelines referring to the long-term health effects after SARS-CoV-2 infection. One explanation for this could be the time at which the included studies collected their data: 11 of the included studies were based on data from the first year of the pandemic when the long-term health effects of acute SARS-CoV-2 infection were only just being observed and definitions, such as those of the NICE guideline and the WHO, had not yet been published. The inconsistent use of standardized evidence-based definitions led to analyses of different time periods in which present symptoms were analysed. Studies that measured symptoms earlier than 4 weeks after acute COVID-19 infections carry a high risk of misclassifying symptoms of acute infections as long-term symptoms. According to the WHO definition and the NICE guideline, long-term symptoms may not only persist after acute infections, but they may also newly develop within 12 weeks of disease onset. Studies that measured symptoms earlier than 12 weeks carry the risk of missing long-term symptoms that newly developed within 12 weeks after the initial infection.

The analysed SEP measures education, employment and income, which are commonly used to describe social inequalities in health outcomes, are limited in their description of socio-economic position: e.g., income does not give any information on wealth or insurance coverage, the socio-economic position does not rise linearly with years of education, and the majority of the used measures of employment like “retirees” or “self-employees” do not describe socio-economic position [60]. Although race/ethnicity is sometimes used as proxy for socio-economic position in epidemiological research, the concept of race/ethnicity is much more complex. It is essential to consider that inequalities in health may be caused by different structural disadvantages minorities face beyond their socio-economic position [61]. This must be considered when implementing public health measures to address possible inequalities in long COVID/PCC. No study was found in the systematic literature search that investigated the associations between socio-economic inequalities and post-COVID-19 conditions as the primary research objective. Consequently, no study was specifically designed to address the research question. Some of the included studies exhibited issues with the representativeness of the general population, small sample sizes and variations in their definitions of long-term health effects of COVID-19. By presenting associations between social determinants and long-term health effects of COVID-19 some studies encountered the risk of a Table 2 fallacy [62].

The current study provides an initial structured overview of the association between the social inequalities and long-term health effects of COVID-19. By conducting a systematic literature search using the Embase and Scopus databases, we ensured a thorough analysis, as these databases cover a wide range of biomedical and social science literature. However, it is possible that potentially relevant literature is not covered by them. To mitigate this, we additionally searched the reference lists of the included articles manually. Furthermore, by focussing on the general population we might have systematically excluded minority populations or populations, which are considered “hard to reach,” such as homeless people or refugees. We considered this search strategy appropriate to address our objectives, i.e., drawing a general comprehensive picture of social inequalities in long-term health effects of COVID-19. However, more in-depth research with an appropriate study design is needed here. We limited the systematic search to peer-reviewed articles to ensure a certain quality of the included articles, as risk of bias assessment was not possible due to the pronounced heterogeneity of the studies. However, the absence of a systematic risk of bias assessment and the limitation to peer-reviewed studies may have led to biased results.

The present review identified important gaps in the research into long COVID/PCC and may help to guide future studies. Further research into long COVID/PCC must be based on standardized clinical case definitions or guidelines with clear time measurement to ensure that long-term symptoms are not confused with symptoms of acute infection or that long-term symptoms have had time to develop. Additionally, it must be ensured that measured symptoms do not result from other health issues, which is a challenge in research into long COVID/PCC, as most sequelae of COVID-19 are unspecific [63, 64]. The risk of misclassification can be reduced by including non-COVID-19 control individuals in research into long COVID, which studies investigating the prevalence or characteristics of prolonged symptoms, or the protective effects of vaccines, have previously done [65].

In conclusion, our knowledge and understanding of health inequalities in long COVID/PCC is still limited. Further research is needed as a better understanding of social inequalities might help to guide future public health interventions and health systems planning. For example, it seems to be likely that individuals living in more deprived areas in the UK are more affected by long COVID/PCC than those living in less deprived areas. In general, targeted public health interventions, such as tailored health information and vaccination campaigns in cooperation and with the participation of local communities might be helpful in reducing health inequalities. At the same time, specialized health services could be implemented, particularly in the hardest hit areas. More high-quality studies are needed to extend our knowledge on how the social determinants of health probably influence the distribution of long COVID/PCC across societies in order to be able to implement effective public health and health services interventions.
