# Supplementary material for: Social Inequalities in Long-Term Health Effects After COVID-19—A Scoping Review
Source: Int J Public Health. 2024 Feb 7;69:1606739. doi: 10.3389/ijph.2024.1606739 (PMC10878999; doi:10.3389/ijph.2024.1606739)
Supplement: Supplementary file 1 [file DataSheet1.docx]

SUPPLEMENTARY MATERIAL

**Social inequalities in long-term health effects after COVID-19 – A scoping review**

Nina Lammers^1^, Florian Beese^1^, Jens Hoebel^1^, Christina Poethko-Müller^1^, Benjamin Wachtler^1^

^1^ Department of Epidemiology and Health Monitoring, Robert Koch Institute, Berlin

**Embase**

'long covid'/exp OR 'long covid' OR 'post-covid':ab,ti OR 'long-haul':ab,ti OR 'long-hauler':ab,ti OR 'chronic-covid':ab,ti OR 'post-acute-sequelae':ab,ti OR 'pasc':ab,ti OR ('persistent-symptom':ab,ti AND 'covid 19') OR (('long-term sequel':ab,ti OR 'longterm sequelae':ab,ti OR 'long-term sequelae':ab,ti) AND 'covid 19') OR (fatigue:ab,ti AND 'covid 19') OR (('long term':ab,ti OR longterm:ab,ti) AND 'covid-19 symptoms':ab,ti) OR (('long term':ab,ti OR longterm:ab,ti) AND 'covid symptoms':ab,ti) OR (('long term':ab,ti OR longterm:ab,ti) AND 'covid-19':ab,ti) OR ('post covid 19' AND condition:ab,ti)

AND

'socioeconomics'/exp OR employment:ab,ti OR education:ab,ti OR income:ab,ti OR occupation:ab,ti OR occupational:ab,ti OR 'living condition':ab,ti OR 'deprivation index':ab,ti OR 'deprivation indices':ab,ti OR 'socioeconomic factor':ab,ti OR 'socioeconomic factors':ab,ti OR 'socioeconomic status':ab,ti OR 'socio-economic status':ab,ti OR 'socioeconomic inequality':ab,ti OR 'socio-economic inequality':ab,ti OR 'socioeconomic inequalities':ab,ti OR 'socio-economic inequalities':ab,ti OR 'socioeconomic differences':ab,ti OR 'socio-economic differences':ab,ti OR 'socioeconomic determinants':ab,ti OR 'socio-economic determinants':ab,ti OR 'socioeconomic gradient':ab,ti OR 'socio-economic gradient':ab,ti OR 'socioeconomic gradients':ab,ti OR 'socio-economic gradients':ab,ti OR 'social position':ab,ti OR 'social-class':ab,ti OR 'health-inequality':ab,ti OR 'health inequalities':ab,ti OR 'health-disparity':ab,ti OR 'health-disparities':ab,ti

AND

([english]/lim OR [german]/lim)

AND

[2020-2022]/py

**Scopus**

( TITLE-ABS

( ( "long-covid"  OR  "post-Covid"  OR  "long-haul"  OR  "longhauler"  OR  "long-hauler"  OR  "chronic-COVID"  OR  "post-acute-sequelae"  OR  "pasc"  OR  ( "persistent-symptom"  AND  "Covid-19" )  OR  ( ( "long-term sequel"  OR  "longterm sequelae"  OR  "long-term sequelae" )  AND  "COVID-19" )  OR  ( "fatigue"  AND  "COVID-19" )  OR  ( ( "long-term"  OR  "longterm" )  AND  "Covid-19 symptoms" )  OR  ( ( "long-term"  OR  "longterm" )  AND  "Covid symptoms" )  OR  ( ( "long-term or longterm" )  AND  "Covid-19" )  OR  "post-COVID-19 AND condition"  OR  "post COVID 19 condition" ) ) )

AND

( TITLE-ABS ( ( "socioeconomics"  OR  "employment"  OR  "education"  OR  "income"  OR  "occupation"  OR  "occupational"  OR  "living condition"  OR  "deprivation index"  OR  "deprivation indices"  OR  "socioeconomic factor"  OR  "socioeconomic factors"  OR  "socioeconomic status"  OR  "socio-economic status"  OR  "socioeconomic inequality"  OR  "socio-economic inequality"  OR  "socioeconomic inequalities"  OR  "socio-economic inequalities"  OR  "socioeconomic differences"  OR  "socio-economic differences"  OR  "socioeconomic determinants"  OR  "socio-economic determinants"  OR  "socioeconomic gradient"  OR  "socio-economic gradient"  OR  "socioeconomic gradients"  OR  "socio-economic gradients"  OR  "social position"  OR  "social-class"  OR  "health-inequality"  OR  "health inequalities"  OR  "health-disparity"  OR  "health-disparities" ) ) )

AND

( LIMIT-TO ( PUBYEAR ,  2020 )  OR  LIMIT-TO ( PUBYEAR ,  2021 )  OR  LIMIT-TO ( PUBYEAR ,  2022 ) )

AND

( LIMIT-TO ( LANGUAGE ,  "English" )  OR  LIMIT-TO ( LANGUAGE ,  "German" ) )
